# Elevated THOC5 expression in liver cancer and its implications for tumor progression and therapeutic response

**DOI:** 10.3389/fmed.2025.1596120

**Published:** 2025-08-18

**Authors:** Jin-Shu Pang, Yu Yang, Li Liang, Yu-Jia Zhao, Jin-Dan Li, Li-Peng Li, Xue-Xin Wei, Rui-Zhi Gao, Rong Wen, Yun He, Hong Yang, Xiu-Mei Bai

**Affiliations:** ^1^Department of Medical Ultrasound, First Affiliated Hospital of Guangxi Medical University, Nanning, Guangxi Zhuang Autonomous Region, China; ^2^Guangxi Key Laboratory of Early Prevention and Treatment for Regional High Frequency Tumor, Guangxi Medical University, Nanning, China; ^3^Collaborative Innovation Centre of Regenerative Medicine and Medical Bioresource Development and Application Co-constructed by the Province and Ministry, The First Affiliated Hospital of Guangxi Medical University, Nanning, China

**Keywords:** THOC5, hepatocellular carcinoma, expression, prognosis, ICB therapy

## Abstract

**Purpose:**

Cancer remains a major global cause of death, with rising incidence influenced by environmental factors. The THOC5 gene, part of the THO complex, has emerged as a potential regulator in cancer biology. This study investigates THOC5 expression across various cancers, its role in prognosis, and its potential therapeutic implications, particularly in liver hepatocellular carcinoma (LIHC).

**Methods:**

We analyzed THOC5 expression and its prognostic significance across various cancer types using globally available public datasets. Single-cell RNA sequencing, in-house RNA-seq, immunohistochemistry, and proteomic analysis were employed to further validate THOC5 expression and prognosis in LIHC. Functional assays, including wound-healing and Transwell migration, were performed following THOC5 knockdown in LIHC cell lines. A drug sensitivity analysis was performed to identify therapeutic agents associated with THOC5 expression levels.

**Results:**

Results indicate that THOC5 is overexpressed in various cancers and correlates with unfavorable prognosis. In LIHC, both THOC5 mRNA and protein was confirmed to be overexpressed in LIHC elevated THOC5 correlated with advanced tumor stages and poor survival outcomes. THOC5 knockdown suppressed *in vitro* cell proliferation, migration, and invasion. Additionally, high THOC5 expression was linked to increased immune cell infiltration and enhanced sensitivity to certain chemotherapy agents, though it predicted poor response to immune checkpoint blockade therapy. Furthermore, high THOC5 was also indicated to activate several tumor signaling pathways, such as EGFR, Hypoxia and MAPK pathways.

**Conclusion:**

THOC5 could be a prognostic biomarker and therapeutic target in LIHC and various cancers, providing alternative treatment options when immunotherapy fails.

## 1 Introduction

Cancer significantly contributes to global mortality and poses a substantial obstacle to enhancing human longevity (1, 2). Key risk factors for cancer include smoking (especially cigarettes), excessive alcohol intake, obesity, sedentary lifestyles, diets deficient in fruits and vegetables, infections, and exposure to sunlight (3), et al. Advances in medical science have led to more frequent identification of new cancer cases, consequently elevating the global incidence of cancer (4–6). Recent progress in novel molecular therapies has also notably improved the prognosis for various types of cancer (7–9). However, influenced by factors such as population aging and increased environmental pollution, the incidence of cancer continues to rise and shows a trend toward affecting younger populations (10). Consequently, it is crucial to identify additional therapeutic targets and prognostic indicators for effective cancer management.

In mammals, the THO complex is part of the Transcription-Export (TREX) ribonucleoprotein complex and includes subunits THOC1, THOC2, THOC3, THOC5, THOC6, and THOC7 (11). It functions to couple processes such as transcription to the splicing, extension, and export of nascent RNA (11). THOC2 functions as a scaffold in the THO complex, and THOC5 facilitates the release of spliced mRNA from the nucleus (12). Recent advances in scientific research have increasingly highlighted the significance of THOC5 in tumor biology. Studies *in vitro* have demonstrated that THOC5 is essential for the self-renewal of tumor stem cells, and the knockdown of THOC5 can reduce breast cancer stem-like traits and enhance the sensitivity of tumors to radiotherapy (13). Additionally, data have shown the pivotal role of THOC5 depletion in inducing apoptosis in certain cancer cells, such as liver cancer cells (14). Furthermore, depleting THOC5 in liver and cervical cancer cells results in the downregulation of several genes linked to cancer progression (14, 15). These findings reveal the potential role of THOC5 in regulating tumor-related gene expression and raise the question of whether depletion of THOC5 and its related genes may represent a new strategy in cancer therapy.

This study analyzed THOC5 expression patterns using integrated public pan-cancer data. Additionally, we examined the association between THOC5 expression and patient survival, immune cell infiltration, immune-related markers, and mutational status using computer-based algorithms across diverse cancer cohorts. Furthermore, the expression and biological functionality of THOC5 were confirmed using our in-house experiments, such as RNA sequencing (RNA-seq) profiles, immunohistology, scratch assays, and Transwell assays.

## 2 Materials and methods

### 2.1 High-throughput sequencing and immunohistochemistry staining

In total, 110 tumor and 9 peritumoral human LIHC tissue samples were collected from the First Affiliated Hospital of Guangxi Medical University for high-throughput sequencing analysis by Sangon Biotech Co., Ltd. (Shanghai, China). Additionally, 27 adjacent liver tissue samples were obtained from the GSE101432 and GSE65485 datasets and included in the analysis for the evaluation of THOC5 expression. Finally, gene expression was quantified using the STAR and feature counts tools. Furthermore, immunohistochemistry staining (abcam: ab36084) was performed on 20 pairs of LIHC and adjacent tissues.

### 2.2 Data collection and analysis

TCGA pan-cancer datasets were downloaded and analyzed using the Sangberbox 3.0 tool. The genetic mutation and stemness scores of THOC5 across pancancer datasets, including RNA expression-based (RNAss), DNA methylation-based stemness scores (DNAss), tumor mutational burden (TMB), and microsatellite instability (MSI) were analyzed using Sangberbox 3.0 and cBioPortal.

RNA-seq data and microarrays for global multi-center bulk LIHC and non-LIHC specimens were obtained from repositories such as SRA, ArrayExpress, and GEO. The datasets were retrieved using the search query: (LIHC OR hepatocellular OR hepatic OR liver) AND (tumor OR cancer OR malignancy OR carcinoma OR neoplasm). The bulk RNA-seq data were quantified using TPM and then normalized by applying log (x + 1) to all expression profiles. Expression profiles from datasets on the same platform were combined into an aggregated expression matrix using the ‘sva’ package in R to remove batch effects (16). The evidence-based models used to integrate public datasets were descripted in the part of statistical analysis.

### 2.3 Single-cell RNA-seq analysis for cellular heterogeneity

Using scRNA-seq data from the GSE151530 dataset, we analyzed gene expression and distribution differences among 17,164 malignant and 35,625 non-tumor cells across 30 samples. The cells were categorized into seven primary groups: tumor cells, T cells, B cells, cancer-associated fibroblasts (CAFs), tumor-associated macrophages (TAMs), tumor-associated endothelial cells (TECs), and unclassified cells.

### 2.4 Proteomic analysis

Proteomic data, including 125 LIHC and adjacent liver samples, were downloaded from the Clinical Proteomic Tumor Analysis Consortium (CPTAC) program. The KNN function of the “impute” package in R was used, with the number of neighbors set to 10, to impute missing data. Subsequently, the relationship between THOC5 protein expression and prognostic significance was analyzed.

### 2.5 Potential pathway of THOC5 in LIHC

Gene expression is linked to the signaling activity of pathways. The greater the expression of genes within a specific pathway, the higher the likelihood of its activation. In this study, pathway activity analysis of classical cancer pathways was conducted using bulk transcriptome sequencing data from 110 internal LIHC samples. The PROGENy package (HiOmics Cloud Platform)^1^ leverages publicly available perturbation experiment data to identify core genes within the pathways, thereby determining their activation status (17). Additionally, Gene Set Enrichment Analysis (GSEA) was employed to analyze the GO terms of TCGA samples, further investigating the potential functions of THOC5.

### 2.6 THOC5 levels and drug treatment

We analyzed the expression differences of immune checkpoint-related genes between patients exhibiting high versus low THOC5 levels. The tumor immune dysfunction and exclusion (TIDE) algorithm was utilized to forecast immune therapy responses in LIHC subtypes exhibiting different levels of THOC5 expression. The TIDE algorithm assesses immune escape mechanisms, focusing on CTL dysfunction and immunosuppressive factor-induced rejection. Elevated TIDE scores are indicative of a poor response to immune checkpoint blockade (ICB) therapy (18). THOC5 mRNA levels from TCGA and various drug datasets were evaluated utilizing the OncoPredict package in the R software. Samples were divided into high and low THOC5 expression groups using the median expression value as the threshold (19). The variation in drug sensitivity between these high and low expression cohorts was examined using Wilcoxon statistical tests and illustrated through box plots. Additionally, molecular docking simulations were employed to assess the binding affinity between these compounds and THOC5 (20).

### 2.7 Cell lines, culture, and siRNAs

The Huh7 and MLIHC97-H human LIHC cell lines were obtained from Procell. Huh7 and MLIHC97-H cells were maintained in DMEM with 10% FBS (Gibco, 10099141C) and 1% antibiotics at 37°C in a 5% CO2 atmosphere. Antibodies against THOC5 were purchased from Abcam. Three siRNAs targeting distinct sequences were evaluated for their efficiency in knocking down THOC5, and the most effective siRNA was chosen for this study (Table S2). siRNA transfections were performed using RNATransMate (Sangon, E607402).

### 2.8 Reverse transcription and quantitative real-time PCR (qRT-PCR)

Total RNA was extracted using Trizol reagent (Invitrogen, USA) following the manufacturer’s protocol. Reverse transcription was conducted using the PrimeScript RT Reagent Kit with gDNA Eraser (RR047A) (Takara, Japan). Gene expression fold-changes were calculated by normalizing Ct values to the GAPDH internal control. qRT-PCR was performed in triplicate with a total reaction volume of 20 μL. The GAPDH primers were 5’-CAGGAGGCATTGCTGATGAT-3′ and 5’-GAAGGCTGGGGCTCATTT-3′, while the THOC5 primers were 5’-CAGTGGAATGCCTCCTGTGT-3′ and 5’-GAAGGCTGGGGCTCATTT-3′.

### 2.9 *In vitro* cell experiments

#### 2.9.1 Wound-healing assays

Cells were seeded into six-well plates. A wound was induced by scratching the confluent cell monolayer with a pipette tip and incubating it in a medium with 2% serum for 48 h. Images were captured at 0 h (immediately after scratching) and 48 h for each well.

#### 2.9.2 Transwell assays

In transwell assays, cells were serum-starved and then trypsinized. Huh7 cells (1 × 10^5) were seeded in the upper chamber with serum-free DMEM, and the lower chamber was supplemented with DMEM containing 10% FBS. Cells were allowed to migrate for 24 h at 37°C. MLIHC97-H cells were seeded at a density of 3 × 10^5 in the upper chamber, with 20% FBS added to the lower chamber. Cells were permitted to migrate over a 72-h period. Following incubation, cells that did not migrate were removed from the membrane’s upper surface with a cotton swab, and the membrane inserts were then stained with crystal violet. Cell motility was measured by counting the cells that moved to the membrane’s underside. Each well was imaged using a light microscope at 100 × magnification, and the migrated cells were counted.

### 2.10 Statistical analysis

Expression values for both mRNA and protein were presented as mean ± standard deviation (SD). Group differences were assessed using the independent Student’s t-test or ANOVA. The evidence-based algorithms (‘meta’ package) in R was used to calculate the standardized mean difference (SMD) for mRNA levels of each gene. Genes were considered overexpressed in LIHC if SMD > 0 and *p* < 0.05, and underexpressed if SMD < 0 and *p* < 0.05. A random-effects model was employed for heterogeneity (I^2^) values of 50% or greater, while a fixed-effects model was utilized for I^2^ values below 50%.

## 3 Results

### 3.1 Analysis of THOC5 expression in human cancers

**Figure 1:**
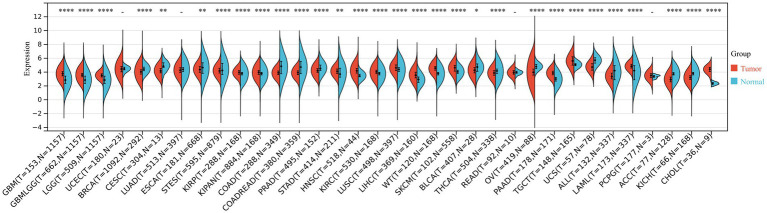
The THOC5 expression levels in tumor and normal tissues across various cancer types based on integrated TCGA and GTEx data. Significant upregulation or downregulation of THOC5 is observed in several cancers, indicating tumor-specific expression patterns.

We systematically analyzed the differential expression of THOC5 across multiple tumor types using integrated data from the cancer genome atlas (TCGA) and genotype-tissue expression (GTEx) cohorts. THOC5 was significantly upregulated in cancers such as glioblastoma multiforme (GBM), lower-grade glioma (LGG), kidney renal papillary cell carcinoma (KIRP), stomach adenocarcinoma (STAD), and liver hepatocellular carcinoma (LIHC), among others, while being downregulated in cancers including breast invasive carcinoma (BRCA), colon adenocarcinoma (COAD), prostate adenocarcinoma (PRAD), and adrenocortical carcinoma (ACC). These results reflect the heterogeneous expression patterns of THOC5 across human malignancies and highlight its potential utility as a diagnostic or therapeutic biomarker (Figure 1).

### 3.2 The relation between THOC5 expression and genetic characteristics

**Figure 2:**
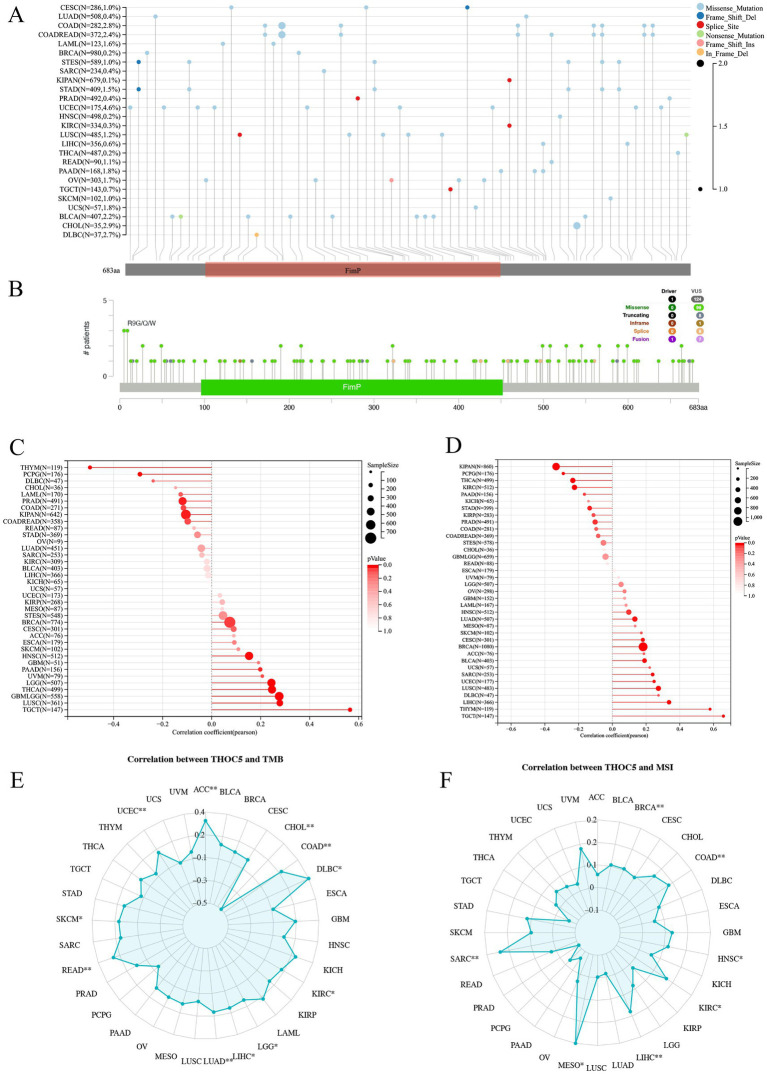
The genomic characteristics and molecular correlations of THOC5 pan-cancer samples. **(A,B)** Mutation frequency and types. **(C,D)** Correlation with tumor stemness (DNAss and RNAss). **(E,F)** Correlation with TMB and MSI. These data suggest that THOC5 may participate in stemness regulation and genomic instability.

As illustrated in Figures 2A,B, somatic mutations of THOC5 were detected in various cancers, with missense mutations being the most frequent type (Figures 2A,B). In the DNAss analysis (Figure 2C), THOC5 expression demonstrated a negative or weak correlation with stemness in cancers such as THYM and LIHC. However, in the RNAss analysis (Figure 2D), THOC5 expression showed a positive correlation with stemness, suggesting that THOC5 may regulate the expression of stemness-related genes. THOC5 expression exhibits significant variation in its correlation with TMB across different cancers. In tumors such as LIHC, LUAD, KIRC, and COAD, THOC5 shows a positive correlation with TMB (Figure 2E), suggesting that THOC5 may contribute to genomic instability and the accumulation of mutations in these cancers. Additionally, correlation analysis between THOC5 and MSI reveals significant positive correlations in COAD, LIHC, and KIRC, further suggesting a possible role in regulating tumor evolution and genomic instability (Figure 2F).

### 3.3 THOC5 expression and prognostic significance

**Figure 3:**
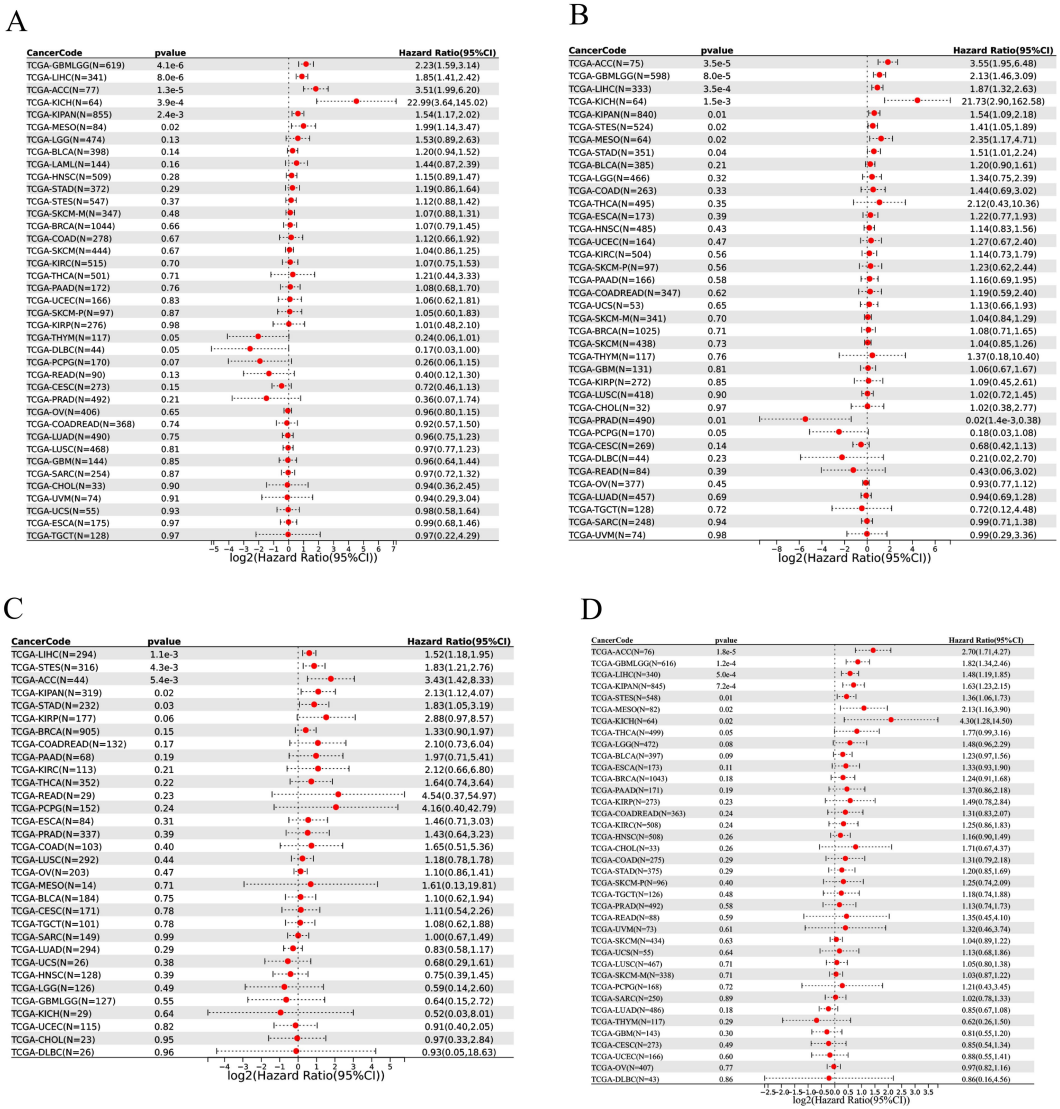
The prognostic value of THOC5 expression across pan-cancer samples. Univariate Cox regression was performed for overall survival (OS, A), disease-specific survival (DSS, B), disease-free interval (DFI, C), and progression-free interval (PFI, D). High THOC5 expression is associated with unfavorable prognosis in multiple tumor types.

To evaluate the prognostic relevance of THOC5, we performed univariate Cox regression analyses covering over 30 cancer types across four clinical endpoints: overall survival (OS), disease-specific survival (DSS), disease-free interval (DFI), and progression-free interval (PFI). High THOC5 expression was significantly associated with poorer outcomes in multiple tumors, particularly GBM, LGG, LIHC, ACC, and KICH, across all four survival indicators (Figure 3). These findings highlight THOC5 as a potential pan-cancer prognostic biomarker.

### 3.4 Elevated expression of THOC5 and its prognostic value in LIHC

**Figure 4:**
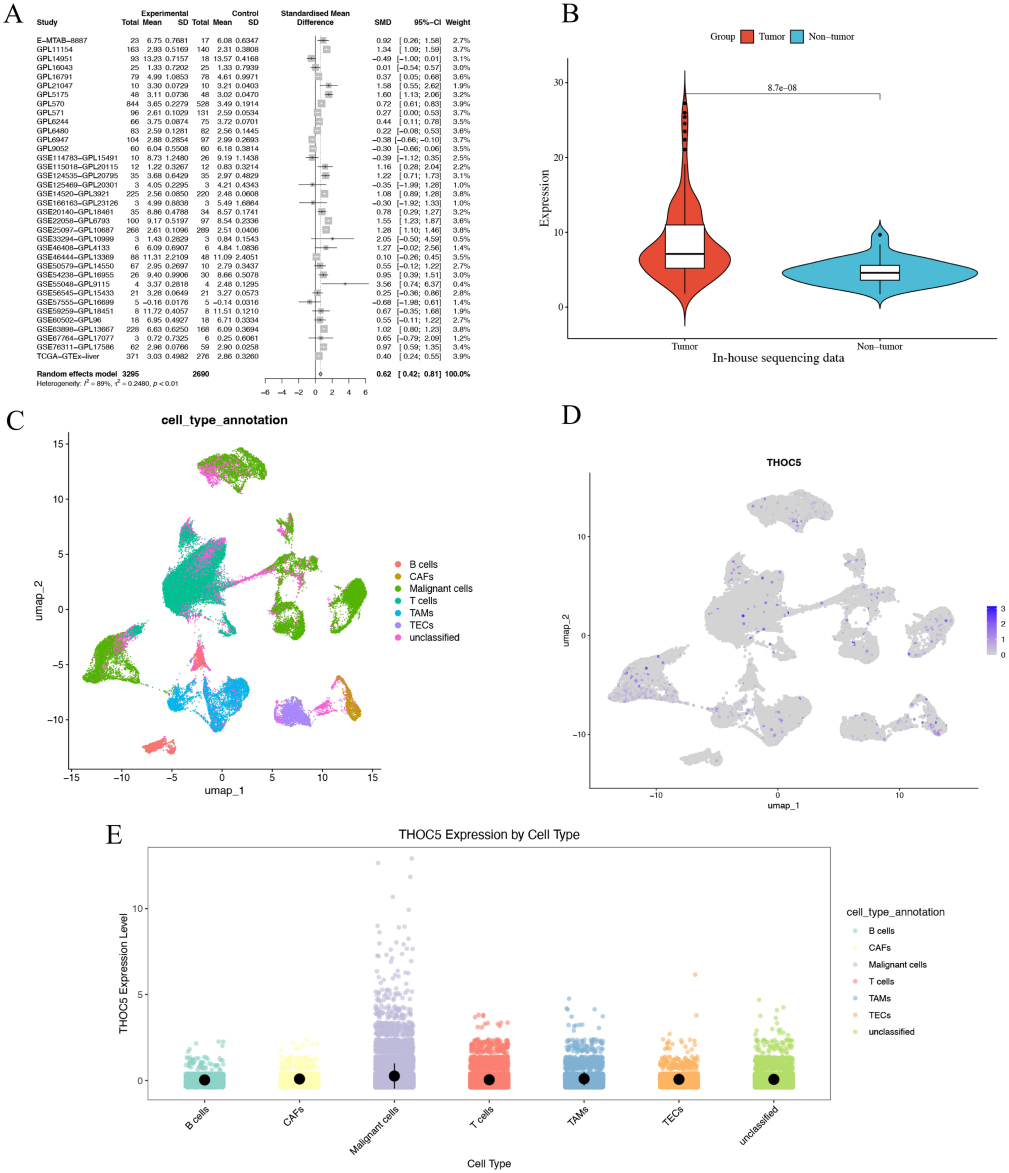
The expression pattern of THOC5 mRNA in LIHC. **(A)** The random forest plot illustrates a significant overexpression of THOC5 in LIHC with 3,295 cases of tumors and 2,690 cases of non-tumor livers. **(B)** In-house sequencing data validates the high expression of THOC5 in LIHC with 110 cases of tumors and 36 cases of non-tumor livers. **(C–E)** Single-cell sequencing analysis reveals that THOC5 expression is primarily distributed in tumor cells.

**Figure 5:**
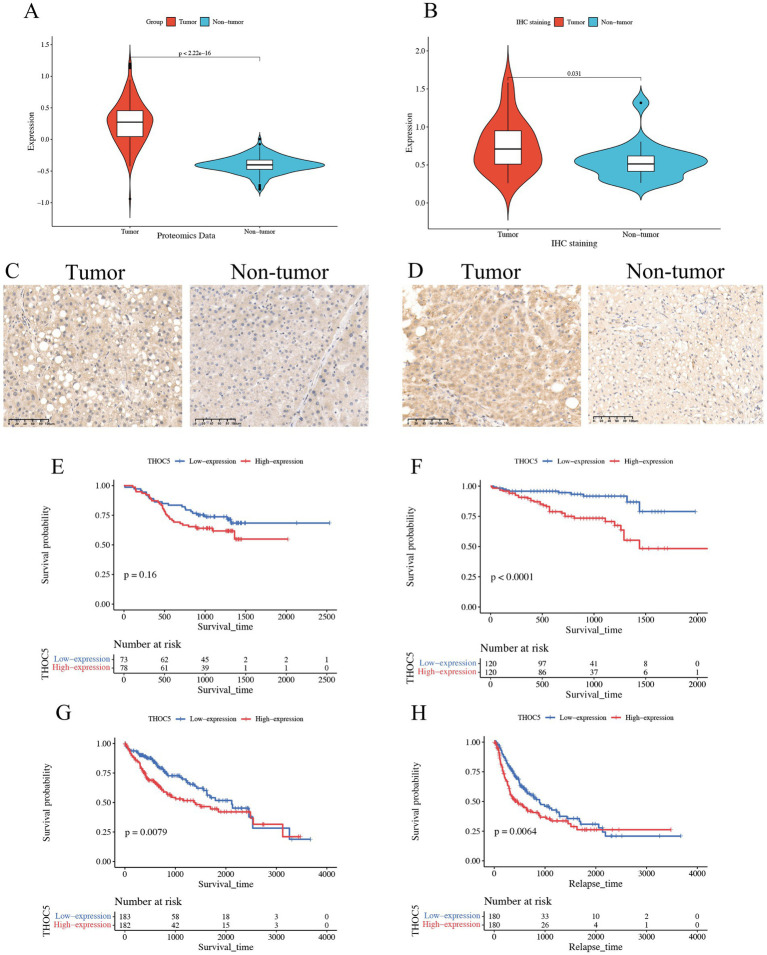
Expression pattern of THOC5 protein and its prognostic value in LIHC. **(A)** CPTAC proteomics analysis shows significantly higher levels of THOC5 protein in HCC compared to adjacent normal tissue; **(B)** immunohistochemical analysis of 20 pairs of cancer and adjacent tissues reveals high expression of THOC5 in the cancer group; **(C,D)** representative images of THOC5 staining in LIHC and adjacent tissues; **(E)** overall survival analysis of THOC5 protein in LIHC using the CPTAC cohort; **(F)** overall survival analysis of THOC5 mRNA in LIHC using the ICGA cohort; **(G)** overall survival analysis of THOC5 mRNA in LIHC using the TCGA cohort; **(H)** disease-free survival analysis of THOC5 mRNA in LIHC using the TCGA cohort.

Given the significant role of THOC5 in LIHC, we aimed to thoroughly investigate its expression and impact on this disease. In total, 3,295 case of cancers and 2,690 non-tumor livers were included to evaluation the expression of THOC5 (Table S1). The global public datasets revealed significant overexpression of THOC5 mRNA in LIHC tissues, with a standardized mean difference (SMD) of 0.62 (95% CI 0.42–0.81), derived from 3,295 tumor and 2,959 non-tumor liver samples (Figure 4A). Our in-house sequencing data confirmed the increased expression of THOC5 in LIHC relative to non-tumor liver tissues (Figure 4B). Single-cell sequencing further categorized liver cancer cells into six clusters, revealing that malignant cells exhibit higher THOC5 expression levels (Figures 4C–E). Furthermore, proteomic analysis and immunohistochemistry (IHC) staining confirmed the elevated THOC5 protein levels in LIHC samples (Figures 5A–D). Collectively, these datasets provide a comprehensive and representative overview of THOC5 expression across a broad global patient population. Multiple cohorts have identified THOC5 as a potential prognostic biomarker for overall survival and disease-free survival in LIHC (Figures 5E–H). High THOC5 expression was also associated with advanced tumor stages, poor tumor grades, and TP53 mutations (Supplementary Figure S1).

### 3.5 THOC5 knockdown inhibited the growth, migration, and invasion of LIHC cells

**Figure 6:**
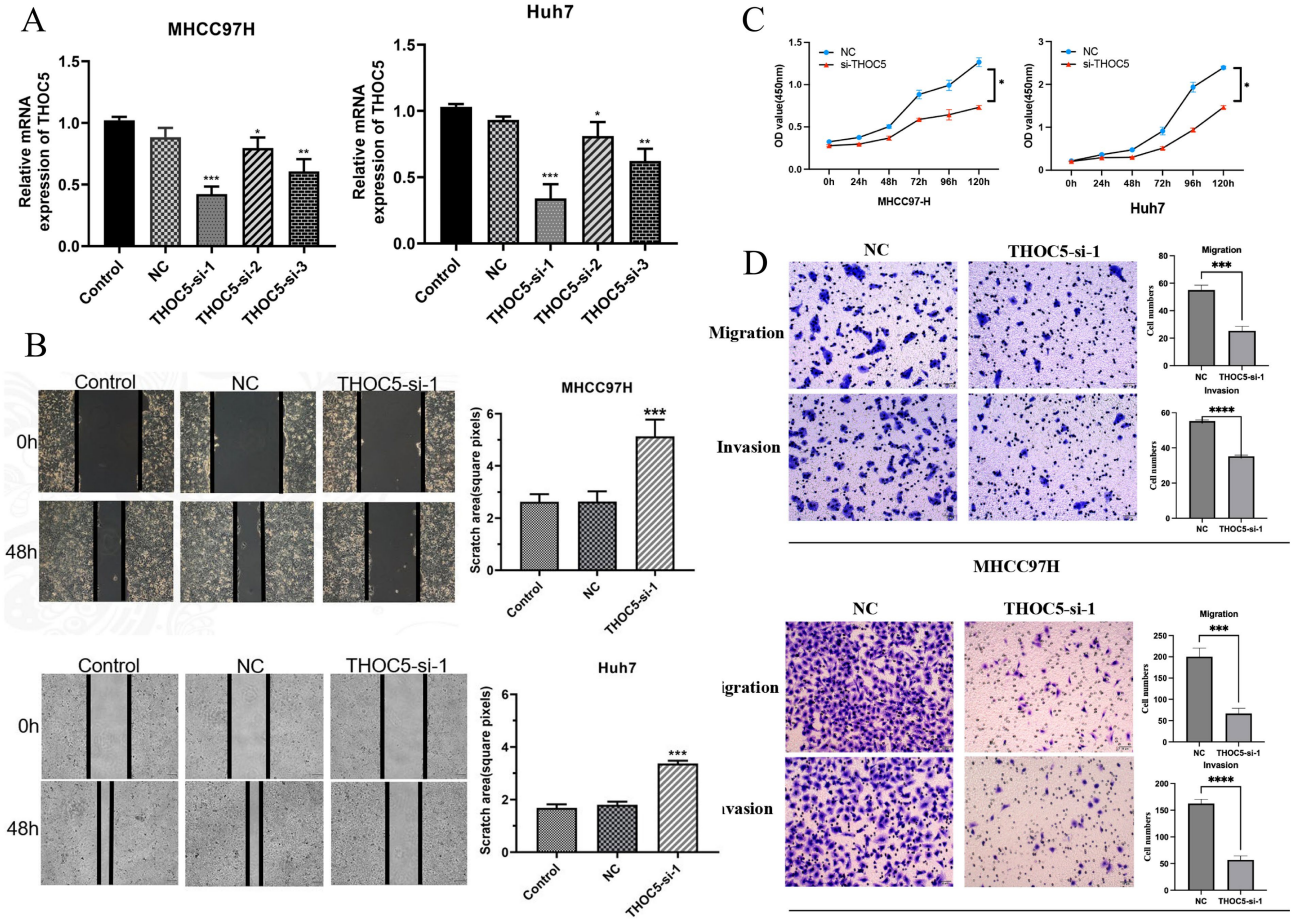
Effect of THOC5 knockdown on liver cancer cells. **(A)** THOC5 mRNA levels in MHCC97H and Huh7 cells after siRNA transfection; **(B)** cell scratch assays show reduced migration capability of MHCC97H and Huh7 cells following THOC5 knockdown; **(C)** CCK-8 assays reveal decreased proliferation of MHCC97H and Huh7 cells after THOC5 knockdown; **(D)** transwell migration and invasion assays demonstrate reduced migration and invasion capabilities of MHCC97H and Huh7 cells following THOC5 knockdown.

After a 48-h transfection with THOC5 siRNA, quantitative real-time PCR (qRT-PCR) confirmed a marked reduction in THOC5 mRNA, indicating the successful knockdown of THOC5 expression in both Huh7 and MHCC97H cell lines (Figure 6A). Functional assays demonstrated that silencing THOC5 significantly reduced LIHC cell viability and suppressed key tumor behaviors, including cell proliferation, migration, and invasion (Figures 6B–D).

### 3.6 The potential mechanism of THOC5 in LIHC

**Figure 7:**
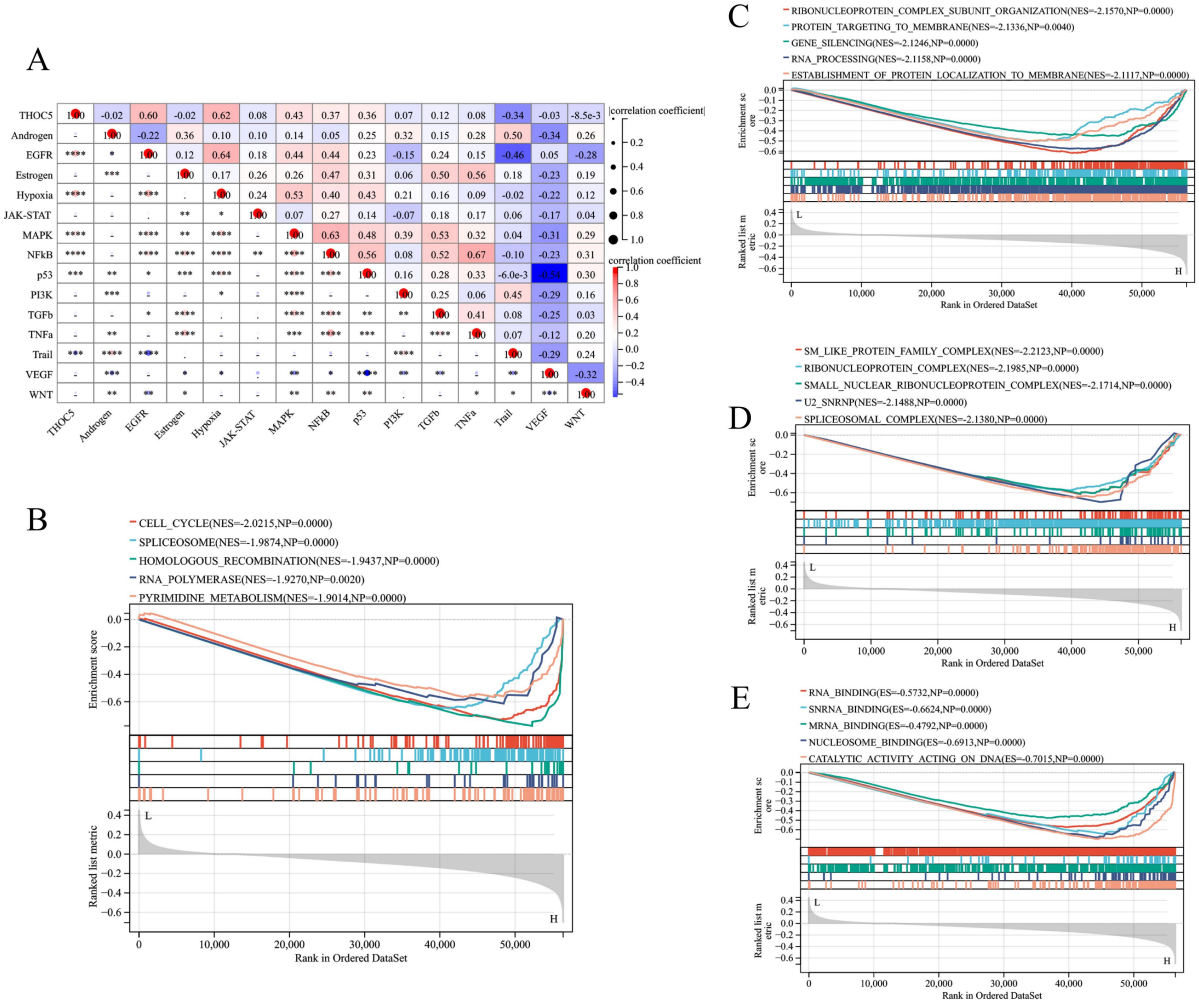
The pathway activities and GESA analysis related to THOC5. **(A)** The correlation between pathway activities and THOC5 expression; **(B)** the KEGG pathway **(C)** biological process, **(D)** cellular component and **(E)** molecular function in the high- and low-THOC5 groups.

According to Figure 7A, high THOC5 expression was also indicated to activate several tumor signaling pathways, such as EGFR, hypoxia, and MAPK pathways. KEGG pathway analysis indicated that THOC5 is linked to pathways including the cell cycle, spliceosome, and homologous recombination, suggesting its potential involvement in transcription regulation, DNA repair, and cellular functions (Figure 7B). Biological process (BP) analysis linked THOC5 expression to RNA processing, protein localization, and gene silencing, crucial for post-transcriptional regulation (Figure 7C). Cellular component (CC) analysis identified significant enrichment in RNA-binding and splicing-related complexes, suggesting THOC5 may influence RNA processing and splicing (Figure 7D). Furthermore, the analysis of molecular function (MF) revealed a significant association between THOC5 expression and key biological processes, including RNA binding, nucleosome binding, and catalytic activity (Figure 7E).

### 3.7 Potential therapeutic implications for LIHC patients with high THOC5 expression

**Figure 8:**
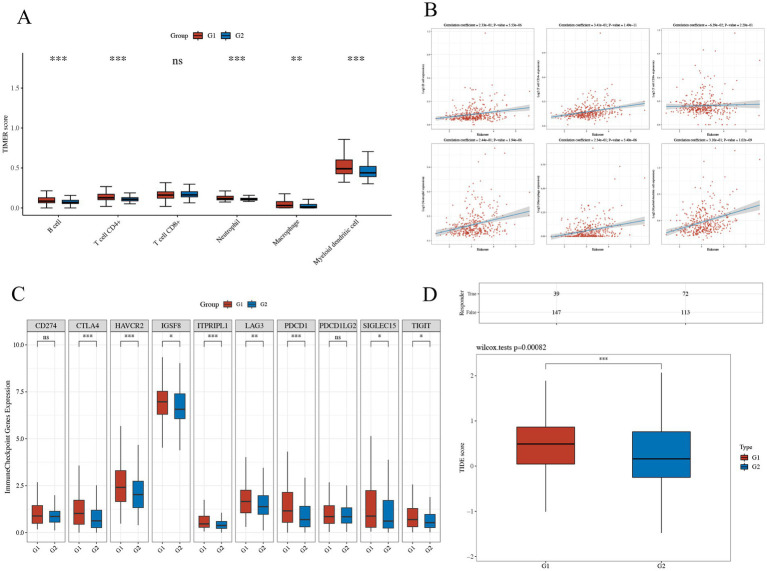
The relations of THOC5 and immune infiltration, inhibitory immune checkpoints, and ICB response. **(A,B)** Correlation between THOC5 expression and the infiltration levels of immune cells; **(C)** expression of immune checkpoints in different THOC5 expression groups; **(D)** TIDE scores predict a low potential response to immunotherapy in the high THOC5 expression group (G1 represents the high THOC5 group and G2 represents the low THOC5 group).

**Figure 9:**
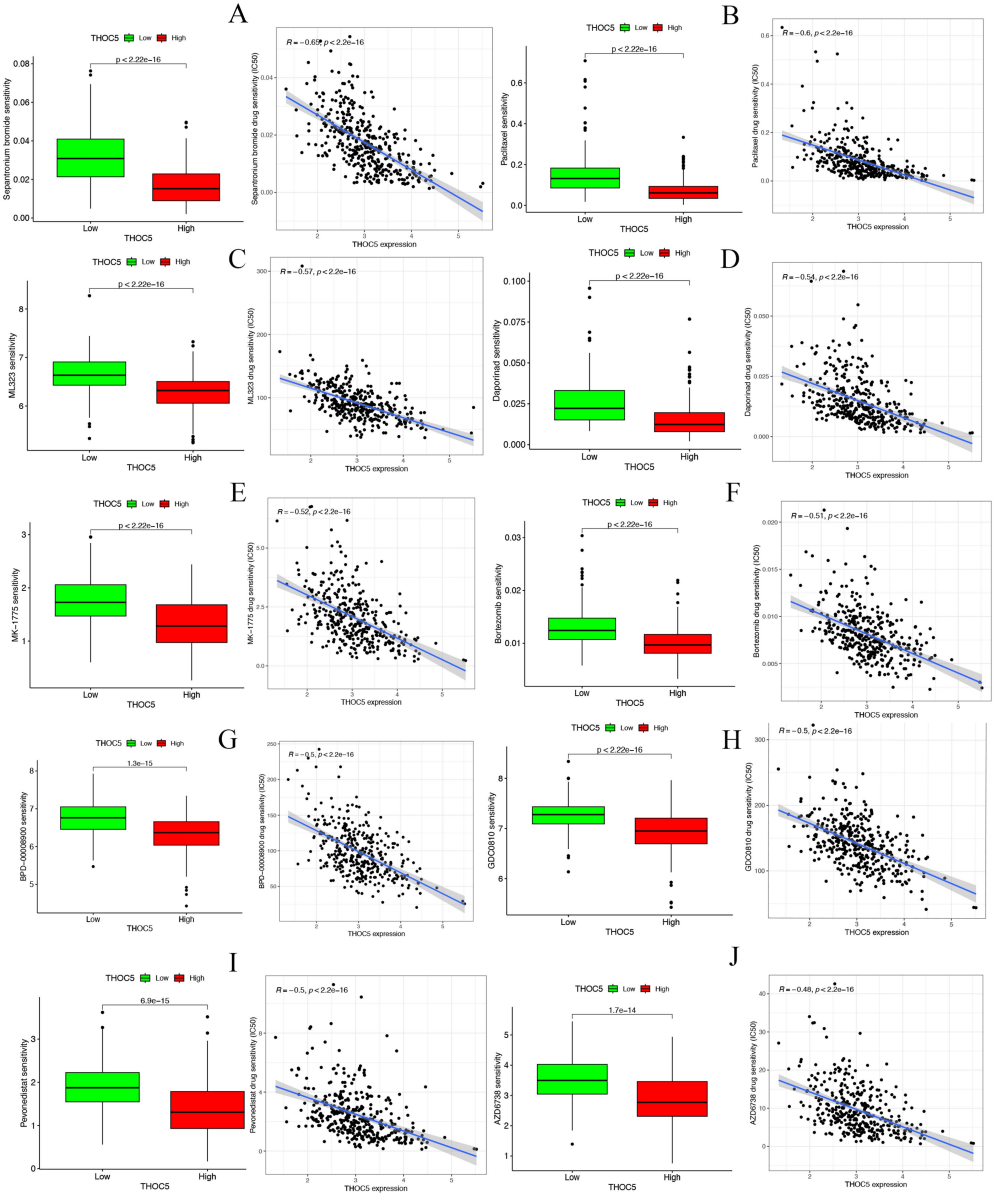
The correlation of THOC5 with anticancer drug sensitivity. **(A)** Sepantronium bromide; **(B)** paclitaxel; **(C)** ML323; **(D)** daporinad; **(E)** MK − 1775; **(F)** bortezomib; **(G)** BPD − 00008900; **(H)** GDC0810; **(I)** pevonedistat; **(J)** AZD6738.

**Figure 10:**
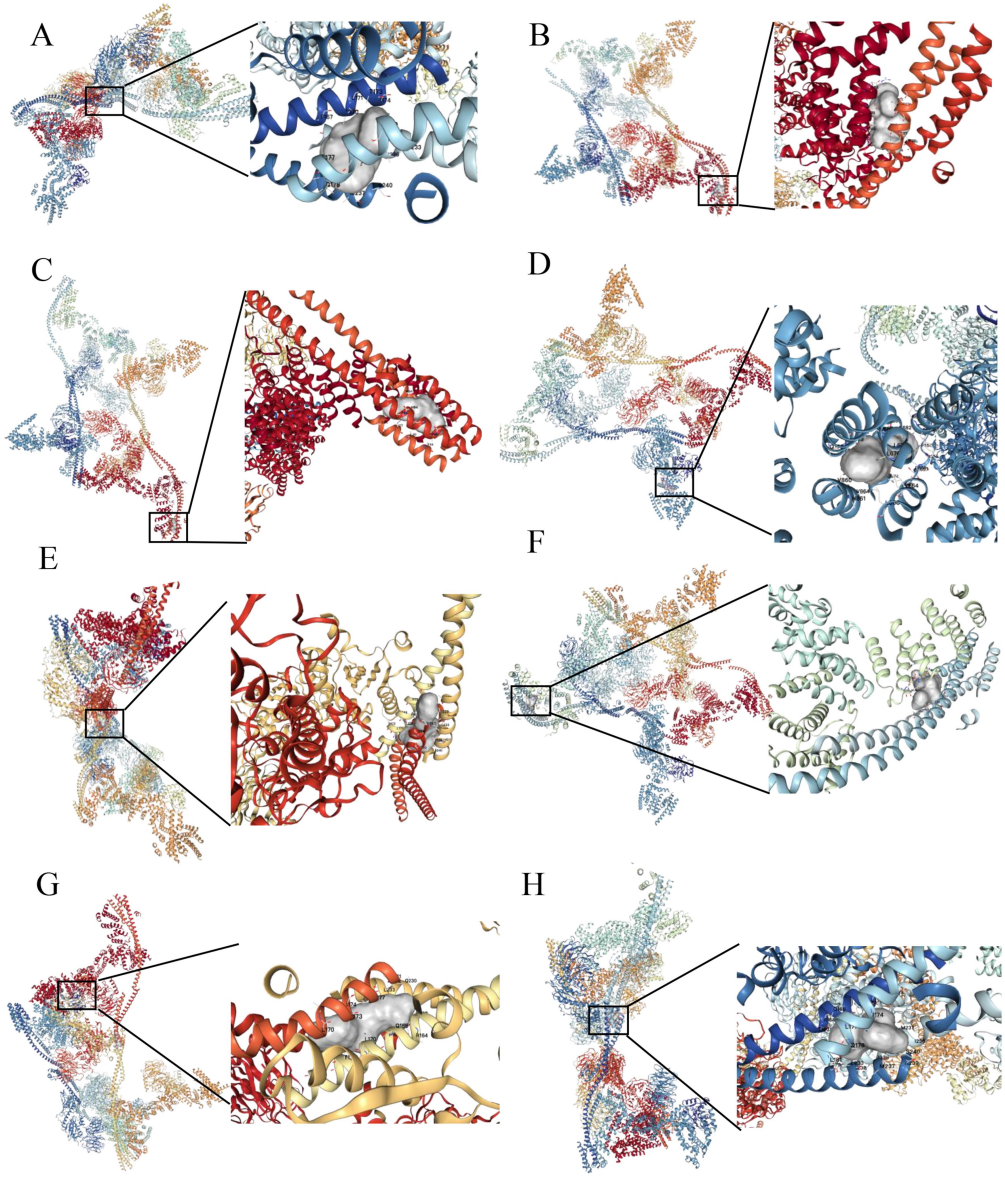
Molecular docking analysis of THOC5 with the top 10 anticancer agents. **(A)** Sepantronium bromide (Vina score = −7.6); **(B)** paclitaxel (Vina score = −8.0); **(C)** ML323 (Vina score = −9.3); **(D)** daporinad (Vina score = −8.8); **(E)** MK − 1775 (Vina score = −9.3); **(F)** GDC0810 (Vina score = −8.7); **(G)** pevonedistat (Vina score = −9.4); **(H)** AZD6738 (Vina score = −8.4). (1) no binding sites were identified between THOC5 and bortezomib, and (2) docking analysis could not be performed for BPD-00008900 due to the unavailability of its structural data in the PubChem database.

The group with elevated THOC5 expression showed significantly increased infiltration of B cells, CD4^+^ T cells, neutrophils, macrophages, and myeloid dendritic cells compared to the group with lower expression (Figure 8A). Furthermore, there was a positive correlation between THOC5 expression intensity and these cell types (Figure 8B). These correlations indicate that THOC5 could significantly influence immune cell distribution and function in the tumor microenvironment. Analysis of immune checkpoint gene expression indicated significant upregulation of most genes, such as PDCD1, CTLA4, and LAG3, in the high THOC5 expression group, with the exception of CD274 and PDCD1LG2 (Figure 8C). This suggests a potential association between high THOC5 expression and immune checkpoint activation. Additionally, the TIDE score was significantly elevated in the high THOC5 expression group, implying a poorer response to immune checkpoint blockade (ICB) therapy (Figure 8D). Considering the limited response of patients with high THOC5 expression to immunotherapy, we conducted a comprehensive drug response analysis using transcriptome data from patients with varying THOC5 expression levels. The results demonstrated that patients in the high THOC5 expression group exhibited significantly increased sensitivity to several traditional or novel antitumor agents, including paclitaxel, sepantronium bromide, docetaxel, YK-4-279, daporinad, AZD6738, vinblastine, MK-1775, ML323, and pevonedistat (Figure 9; Supplementary Table S3). Molecular docking analysis further supported these findings, revealing strong binding affinities between THOC5 and these compounds (Vina score ≤ − 7.0, Figure 10).

## 4 Discussion

Epigenetic abnormalities, such as gene mutations and changes in gene expression, can occur under specific conditions. These changes may lead to the production of excessive or abnormal proteins, which can drive uncontrolled cell growth and metastasis. In cancer biology, oncogenes are genes that, when mutated or overexpressed, contribute to the development and progression of cancer (21). These genes play key roles in disrupting normal cellular processes, which are linked to cancer. Such processes include promoting cell division, preventing programmed cell death, stimulating angiogenesis, and activating signaling pathways that support tumor growth and metastasis (22). Understanding how genes are differentially expressed in cancer cells is essential for uncovering the mechanisms behind cancer development and progression.

THOC5 is an essential part of the THO complex, which is recruited to mRNA during nuclear RNA processing and facilitates its export to the cytoplasm (23). A previous work has indicated that THOC5 selectively exports transcripts involved in cell growth, differentiation, survival as well as tumor development (15). In cancer, overexpression of THOC5 may change its preference for specific target transcripts. This leads to the selective export of oncogenic mRNAs, which in turn increases their stability and translation, thereby promoting malignant proliferation and tumor progression.

High levels of THOC5 expression are associated with increased tumor proliferation, metastasis, and poor prognosis in various cancers (14, 15). THOC5 promotes the expression of oncogenes and supports cancer cell survival by influencing mRNA stability and export. This contributes to the disruption of pathways that regulate tumor growth and metastasis (14, 15). Due to its role in enhancing oncogenic signaling, THOC5 has become a potential target for cancer therapy, especially for cases that are resistant to treatment.

This study investigated THOC5 expression across 33 prevalent tumors utilizing public databases. We found that THOC5 exhibited distinct expression patterns across different tumors, suggesting a complex regulatory role in cancer. Additionally, THOC5 showed significant genetic variations, with missense mutations being the most common and particularly prevalent in BLCA, UCS, and UCEC. However, missense mutations were relatively rare in LIHC patients (only 2 of 356 cases), suggesting that THOC5 overexpression in LIHC is independent of missense mutations.

Through DNAss and RNAss analysis, we observed potential correlation between THOC5 and stemness characteristics. However, THOC5 is not a transcription factor and cannot directly activate gene transcription. These correlations may reflect THOC5’s regulatory function at the post-transcriptional level, particularly its role in promoting the nuclear export and cytoplasmic accumulation of stemness-related mRNAs. A study on triple-negative breast cancer (TNBC) also supports this mechanism: it found that THOC2, dependent on THOC5, facilitates the export of SOX2 and NANOG transcripts, thereby enhancing stemness features and radioresistance (13). Knockdown of THOC5 significantly reduced the protein expression of these key stemness factors, suggesting that THOC5 enhances their expression not through transcriptional activation but via an export mechanism (13). These results indicate that THOC5 may influence tumor stemness by regulating the post-transcriptional fate of key stemness-related mRNAs. Besides, THOC5 showed a positive correlation with TMB and MSI in tumors such as LIHC and LUAD. This indicates that THOC5 might promote genomic instability and mutation accumulation, driving tumor development.

Further prognostic analysis showed that THOC5 negatively impacted OS, DSS, DFI, and PFI in various cancers. In LIHC, high THOC5 expression was strongly associated with poorer outcomes across all four prognostic indicators. This suggests that THOC5 may serve as a key prognostic biomarker for LIHC. We plan to further investigate the molecular mechanisms of THOC5 in HCC, exploring its role in progression and its potential as a therapeutic target. Our goal is to provide more effective diagnostic and treatment strategies for HCC patients.

Analysis of global public database data revealed a significant upregulation of THOC5 mRNA in LIHC tissues. The SMD was 0.62 (CI: 0.42–0.81), based on 3,295 tumor samples and 2,959 non-tumor liver samples. Our in-house sequencing data confirmed this upregulation in LIHC, with significantly higher levels compared to non-tumor liver tissues. Proteomic analysis and IHC staining further validated the elevated THOC5 protein levels in LIHC samples.

These datasets provide a comprehensive overview of THOC5 expression in a global patient population. Additionally, multiple cohort analyses identified THOC5 as a potential prognostic biomarker for OS and DFS in LIHC. High THOC5 expression was also linked to advanced tumor stages, poor tumor grades, and TP53 mutations. These findings align with the pan-cancer analysis results, underscoring THOC5’s potential role in LIHC progression.

Furthermore, KEGG pathway and BP analysis revealed that THOC5 expression is strongly linked to several key signaling pathways, including the cell cycle, spliceosome, homologous recombination, RNA polymerase, and pyrimidine metabolism. This highlights its potential role in transcriptional regulation, DNA repair, and cellular functions. THOC5 may promote cancer cell proliferation and help maintain genomic stability by regulating the cell cycle and DNA repair, which are crucial for tumor cells (24–26). Meanwhile, MF and CC analysis showed that THOC5 is significantly associated with RNA binding, nucleosome binding, and catalytic activity. It plays a particularly important role in RNA splicing and processing. These processes are vital for cancer cell development. Thus, THOC5 may contribute to the abnormal proliferation and metastasis of cancer cells by influencing these mechanisms (27, 28).

Immune infiltration analysis revealed significantly elevated levels of B cells, CD4^+^ T cells, neutrophils, macrophages, and myeloid dendritic cells in the high THOC5 expression group compared to the low expression group. The expression intensity of THOC5 also positively correlated with these cell types. THOC5 may influence immune cell distribution and function within the tumor microenvironment. Analysis of immune checkpoint gene expression indicated significant upregulation of most genes, such as PDCD1, CTLA4, and LAG3, in the high THOC5 expression group, with the exception of CD274 and PDCD1LG2. This indicates that elevated THOC5 expression is associated with immune checkpoint activation, crucial for tumor immune escape and significant in tumor immunotherapy (29–33).

Moreover, the TIDE score was significantly elevated in the high THOC5 expression group, indicating that these patients had a poor response to ICB therapy. Given the limited response to immunotherapy in patients with high THOC5 expression, we performed a comprehensive drug response analysis using transcriptome data from patients with different THOC5 expression levels. The findings revealed that the group with elevated THOC5 expression exhibited increased sensitivity to various conventional anti-tumor drugs. Several drugs, such as paclitaxel, docetaxel, and AZD6738, have been identified for their potential in treating liver cancer (34–36). The results indicate that these drugs could serve as effective treatment options for patients with elevated THOC5 expression when immunotherapy fails, emphasizing the necessity for further research into their clinical applicability for this patient group.

This study has several limitations. First, most of our analyses are based on bioinformatic data and algorithms, with a lack of deeper mechanistic investigations—such as the identification of direct THOC5 targets via CLIP-seq in LIHC cells, pathway regulation analyses, and rescue experiments using export-deficient THOC5 mutants. Second, although we analyzed a large number of tumor and normal samples, additional cohorts from diverse cancer types and more comprehensive clinicopathological parameters are needed to strengthen these findings. Third, functional validation *in vivo* (e.g., xenograft or genetically engineered mouse models) will be important to confirm THOC5’s role in tumor progression and drug response. Finally, our prognostic associations are based on retrospective datasets, and thus, prospective clinical studies are needed to validate THOC5 as a biomarker in hepatocellular carcinoma.

In summary, THOC5 is an important prognostic biomarker in LIHC. It influences the patient’s immunotherapy response by modulating immune cell infiltration and checkpoint expression within the tumor microenvironment. Moreover, elevated THOC5 expression correlated with increased sensitivity to various anti-tumor drugs. This indicates that THOC5 may serve as a potential target for personalized treatment approaches. Future research should investigate the precise role of THOC5 in immune regulation and drug response in LIHC.

## Data Availability

The datasets presented in this study can be found in online repositories. The names of the repository/repositories and accession number(s) can be found in the article/Supplementary material.

## Footnotes

^1^ https://henbio.com/en/tools

## Glossary

LIHC: liver hepatocellular carcinoma
RNA-seq: RNA sequencing
scRNA-seq: single-cell RNA sequencing
CPTAC: Clinical Proteomic Tumor Analysis Consortium
GSEA: Gene Set Enrichment Analysis
TIDE: tumor immune dysfunction and exclusion
ICB: immune checkpoint blockade
FBS: fetal bovine serum
DMEM: Dulbecco’s modified eagle medium
qRT-PCR: quantitative real-time polymerase chain reaction
SRA: sequence read archive
GEO: gene expression omnibus
TPM: transcripts per million
SD: standard deviation
ANOVA: analysis of variance
SMD: standardized mean difference
I^2^: heterogeneity index
TCGA: the cancer genome atlas
GTEx: genotype-tissue expression
GBM: glioblastoma
LGG: lower-grade glioma
KIRP: kidney renal papillary cell carcinoma
KIPAN: kidney pan-cancer
STAD: stomach adenocarcinoma
HNSC: head and neck squamous cell carcinoma
KIRC: kidney renal clear cell carcinoma
LUSC: lung squamous cell carcinoma
WT: Wilms’ tumor
SKCM: skin cutaneous melanoma
PAAD: pancreatic adenocarcinoma
TGCT: testicular germ cell tumors
LAML: acute myeloid leukemia
CHOL: cholangiocarcinoma
BLCA: bladder urothelial carcinoma
UCS: uterine carcinosarcoma
UCEC: uterine corpus endometrial carcinoma
DNAss: DNA stemness
RNAss: RNA stemness
TMB: tumor mutational burden
MSI: microsatellite instability
OS: overall survival
DSS: disease-specific survival
DFI: disease-free interval
PFI: progression-free interval
KEGG: Kyoto Encyclopedia Of Genes And Genomes
BP: biological process
CC: cellular component
MF: molecular function
IHC: immunohistochemistry
