# Supplementary figures and images for: Elevated THOC5 expression in liver cancer and its implications for tumor progression and therapeutic response
Source: Front Med (Lausanne). 2025 Aug 18;12:1596120. doi: 10.3389/fmed.2025.1596120 (PMC12400153; doi:10.3389/fmed.2025.1596120)

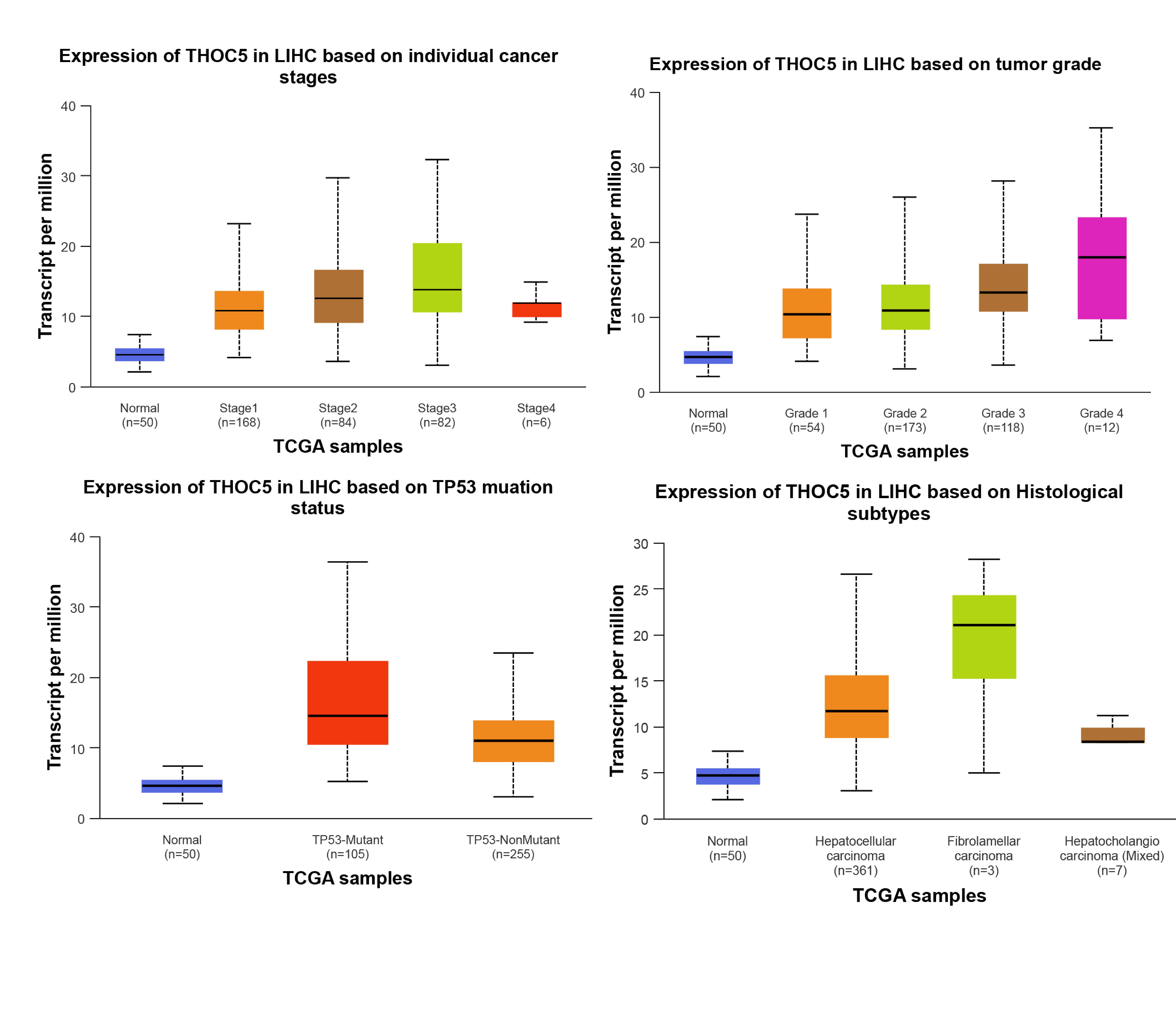

Supplement: Supplementary file 1 [file Image_1.jpeg]
