# Supplementary material for: Elevated THOC5 expression in liver cancer and its implications for tumor progression and therapeutic response
Source: Front Med (Lausanne). 2025 Aug 18;12:1596120. doi: 10.3389/fmed.2025.1596120 (PMC12400153; doi:10.3389/fmed.2025.1596120)
Supplement: Supplementary file 3 [file Table_2.docx]

Table S1 The siRNAs for THOC5 knockdown in this study

| RNA Name | | 5’-3’ Sequences |
| --- | --- | --- |
| THOC5-si-1 | Forward- CCAGCCAAUCAGUAUCAGUUUT  Reverse- AAACUGAUACUGAUUGGCUGGTT | |
| THOC5-si-2 | Forward- CUACAGAAGGAGAUCACCAAATT  Reverse- UUUGGUGAUCUCCUUCUGUAGTT | |
| THOC5-si-3 | Forward- GAUGUGGCAAUAGAAAUAGAATT  Reverse- UUCUAUUUCUAUUGCCACAUCTT | |
| NC | Forward- UUCUCCGAACGUGUCACGUTT  Reverse- ACGUGACACGUUCGGAGAATT | |
