# Supplementary material for: Elevated THOC5 expression in liver cancer and its implications for tumor progression and therapeutic response
Source: Front Med (Lausanne). 2025 Aug 18;12:1596120. doi: 10.3389/fmed.2025.1596120 (PMC12400153; doi:10.3389/fmed.2025.1596120)
Supplement: Supplementary file 4 [file Table_3.docx]

Table S3. The Correlation of THOC5 with anticancer drug sensitivity with a |r|> 0.4.

| Drug | Correlation | p-Value |
| --- | --- | --- |
| Sepantronium bromide | -0.65 | p < 2.2e−16 |
| Paclitaxel | -0.60 | p < 2.2e−16 |
| ML323 | -0.57 | p < 2.2e−16 |
| Daporinad | -0.54 | p < 2.2e−16 |
| MK−1775 | -0.52 | p < 2.2e−16 |
| Bortezomib | -0.51 | p < 2.2e−16 |
| BPD−00008900 | -0.50 | p < 2.2e−16 |
| GDC0810 | -0.50 | p < 2.2e−16 |
| Pevonedistat | -0.50 | p < 2.2e−16 |
| AZD6738 | -0.48 | p < 2.2e−16 |
| Tozasertib | -0.48 | p < 2.2e−16 |
| Axitinib | -0.47 | p < 2.2e−16 |
| Telomerase Inhibitor IX | -0.46 | p < 2.2e−16 |
| Vinblastine | -0.46 | p < 2.2e−16 |
| Docetaxel | -0.45 | p < 2.2e−16 |
| MG−132 | -0.45 | p < 2.2e−16 |
| YK−4−279 | -0.45 | p < 2.2e−16 |
| PAK_5339 | -0.43 | p < 2.2e−16 |
| BMS−345541 | -0.42 | p < 2.2e−16 |
| UMI−77 | -0.41 | p < 2.2e−16 |
| Wee1 Inhibitor | -0.41 | p < 2.2e−16 |
| Wnt−C59 | -0.41 | p < 2.2e−16 |
| WIKI4 | -0.40 | p < 2.2e−16 |
| AZD6482 | 0.41 | p < 2.2e−16 |
| Entinostat drug | 0.44 | p < 2.2e−16 |
| Doramapimod | 0.45 | p < 2.2e−16 |
| JAK1_8709 | 0.47 | p < 2.2e−16 |
| Nutlin−3a (−) | 0.47 | p < 2.2e−16 |
